# Changes in the chick liver structure and blood plasma biochemical properties following in ovo administration of acrylamide

**DOI:** 10.1016/j.psj.2026.107580

**Published:** 2026-08-04

**Authors:** J. Wojciechowska-Puchałka, E. Drąg-Kozak, K. Kustra, M. Trela, M.W. Lis

**Affiliations:** a Department of Animal Reproduction, Anatomy and Genomics, University of Agriculture in Krakow, Mickiewicza Alley 24/28, 30-059 Krakow, Poland; b Department of Animal Nutrition and Fisheries, University of Agriculture in Krakow, Mickiewicza Alley 24/28, 30-059 Krakow, Poland; c Department of Zoology and Animal Welfare, University of Agriculture in Krakow, Mickiewicza Alley 24/28, 30-059 Krakow, Poland

**Keywords:** Acrylamide, Liver, Microstructure, Chick, Trace elements

## Abstract

This study analyzed the effects of acrylamide administration during the embryonic period on liver microstructure and blood plasma biochemical parameters in hatched chicks. On day 6 of incubation, 96 embryonated eggs per group were injected with acrylamide (ACR) at doses of 0.0, 0.25, 0.5, 1.0, 2.0 and 4.0 mg ACR/egg dissolved in 0.9% saline solution (100 µL). Embryo development, hatchability and quality of chicks were evaluated. The ten chicks per group (0.0, 0.25, 0.5 and 1.0 mg ACR/egg) were euthanized immediately after hatching, and blood and liver samples were collected for further analysis. Hatchability was 74.0, 67.7, 66.7 and 49.5% in groups treated with 0.0, 0.25, 0.5, 1.0 mg ACR/egg, respectively, while all embryos died in 2.0 and 4.0 mg ACR/egg groups. Acrylamide intoxication affected only a few biochemical parameters of blood, including a significant decrease in glucose and globulin levels in the group injected with a dose of 1 mg/egg and a significant increase in blood urea nitrogen concentration in the group treated with a dose of 0.5 mg/egg. However, this substance caused negative changes in the microarchitecture of the liver of one-day-old chicks, as indicated by the results of histomorphometric analysis, such as an increase in total hepatocyte nuclei, binucleated hepatocytes, cell nucleus size, collagen area, and other cells. Furthermore, abnormalities in liver structure were confirmed by trace elements content results. Thus, in individuals treated with acrylamide (0.25 and 0.5 mg/egg), a decrease in copper levels and (0.25 mg/egg) nickel and iron levels and an increase in cadmium levels were observed. An increase in lead content was similarly observed in the group exposed to acrylamide at a dose of 0.5 mg/egg, as well as an increase in zinc levels in groups administered various doses of this toxic substance. Overall, the results suggest an inconsistent and dose-dependent toxic effect of acrylamide on liver structure and selected blood plasma parameters in one-day-old chicks.

## Introduction

The development of civilization, urbanization and food processing has led to the creation of a number of toxicological hazards that can present a threat to human and animal health and life. One such factor is acrylamide (**ACR**), which is not naturally occurring. It can be formed as a byproduct during the heat treatment of food during the Maillard reaction or can be produced on an industrial scale by hydrolysis of acrylonitrile (Koszucka et al., 2020; Mottram et al., 2002).

Consequently, its presence has been reported in many foods (especially fried, baked and roasted), as well as livestock feed, specifically in mixtures intended for poultry (Halle et al., 2006; Kienzle et al., 2005; Rice, 2005). Importantly, some reports indicate the transfer of ACR from livestock animals to milk and eggs (Halle et al., 2006; Kienzle et al., 2005; Pabst et al., 2005). Thus, Halle et al. (2006) in a study conducted on 16 Lohmann Select Leghorn laying hens, indicate that chronic 4-week dietary exposure of females to this toxic substance (at a dose of 671 ± 32 µg per kg) leads to the transfer of ACR into the egg at a maximum concentration of 17.2 µg/kg of egg weight. Although the biological likelihood of embryonic exposure via the egg is high, the authors did not focus on embryonic development in their experiment and therefore did not examine fertilized eggs. Furthermore, such analyses are still lacking in the available literature.

In addition, as ACR is absorbed not only through the gastrointestinal tract, but also through the respiratory system and skin, ACR intoxication can also result from occupational exposure (e.g. in foundries or production factories) (Friedman, 2003). ACR polymers are used in the production of cosmetics, fertilizers, paper products, contact lenses, textiles, paints, varnishes and also in laboratories (for example for the production of polyacrylamide gels) (Friedman, 2003). Polyacrylamide is also used in wastewater treatment technology and water treatment (Murkovic, 2004). So from a chemical point of view, it is an organic compound very soluble in water, acetone, alcohol, ether, chloroform, but insoluble in benzene and heptanes (Stadler and Scholz, 2004). The substance is a crystalline substance in the form of flakes or crystals without color, smell or taste. When exposed to UV light or heated, ACR polymerises into ACR polymers and copolymers, which have just been used in many industrial and agricultural applications, especially for soil conditioning, erosion control, increasing water retention, and as slow-release carriers for fertilizers and herbicides (Kepekci Tekkeli et al., 2012; Lentz, 2015; Song et al., 2024). Therefore, this substance can be released unintentionally or intentionally into the environment and cause its pollution. Furthermore, data from the literature indicate that ACR is detected in water at low concentrations of up to 17–20 ng/L (Backe et al., 2014; Togola et al., 2015). However, because this substance is highly soluble and very mobile, it can migrate into surface and groundwater, causing contamination not only of the water but also of the soil (Tepe and Çebi, 2019; Wang et al., 2022).

Numerous studies on humans, mammals and birds indicate that ACR has toxic effects on the nervous system, reproductive system or genetic material (Batoryna et al., 2018; Beland et al., 2013; Hułas-Stasiak et al., 2013; Katen and Roman, 2015; Palus et al., 2019; Sun et al., 2018; Yu et al., 2019). In addition, it has been proven that this chemical induces oxidative stress, apoptosis and inflammation. Thus, Naruszewicz et al. (2009), in a human study, confirmed that ACR induces oxidative stress in leukocytes and triggers inflammation, as evidenced by serum markers such as hs-CRP and hs-IL-6. Meanwhile, Sengul et al. (2021) demonstrated that this toxic substance exacerbates oxidative stress, inflammation, and apoptosis in the kidneys in male Sprague-Dawley rats. Furthermore, Yousef and El-Demerdash (2006) confirmed that ACR induces oxidative stress in the plasma, liver, testes, brain, and kidneys, and induces enzymatic damage in the plasma, liver, testes, and brain of adult Sprague–Dawley rats. Furthermore, studies indicate that exposure to ACR in various forms (ingestion or inhalation) leads to accumulation of this substance primarily in the blood (Shipp et al., 2006). Hence, as early as 1994, the International Agency for Research on Cancer (IARC) recognized ACR as a substance with mutagenic, teratogenic and neurotoxic effects (Group 2A). In addition, European Commission Regulation No. 2158/2017 of November 20, 2017 recognizes ACR as a mutagenic substance of particular danger in food. Unfortunately, no limit for acceptable ACR doses in food has been established to date, only benchmark levels for various categories of food products (e.g., 750 µg/kg for potato chips, 500 µg/kg for French fries, and 40 µg/kg for infant cereal products). However, in 2009, the FAO/WHO issued the „Code of Practice for the Reduction of Acrylamide in Foods (CAC/RCP 67-2009)”. A year later, the European Chemicals Agency (ECHA) recognized ACR as a substance of very high concern.

It is well known that the liver is one of the main organs where biotransformation of toxic compounds of endogenous and exogenous origin takes place. The liver is also involved in most metabolic changes, influences the body's homeostasis, and produces almost all blood coagulation factors and coagulation inhibitory factors (protein C and protein S). However, the available literature lacks information on changes in the concentration or activity of these proteins under the influence of ACR, although it is known that this compound can lead to liver damage (Quasmi et al., 2025; Zhang et al., 2023). Therefore, it can be assumed that more severe liver damage caused by ACR poisoning could secondarily affect the synthesis of liver-dependent proteins (such as proteins C and S) and thus contribute to thrombosis (Bereczky et al., 2010). In addition, liver is a very important store of lipids, glycogen, elements (such as iron) or vitamins (B12, B, D, K, A and C). Very importantly, this organ is also responsible for detoxification of the body either by reacting ACR with glutathione (**GSH**) or by converting it to the more toxic epoxide derivative glycidamide (**GA**) with the involvement of cytochrome P450 2E1 (Katen and Roman, 2015; Parzefall, 2008; Stadler and Scholz, 2004). An experiment conducted by Machala et al. (1996) on the ontogenesis of chicken embryos confirms that this enzyme is already present during embryogenesis, although its levels are low at the beginning of this process. However, these studies do not confirm its metabolic activity at such an early stage of embryonic development, which makes it difficult to determine unequivocally whether it enables the biotransformation of ACR to glycidamide. Furthermore, toxic factors including ACR lead to liver damage and induce irreversible changes in liver structure such as fibrosis of the extracellular matrix, increased inflammatory cell infiltration and consequent loss of communication of hepatocytes with blood vessels (Friedman, 2008; Wynn, 2008). This chemical also affects the levels of liver function enzymes and modifies metabolites in the liver and serum (Cengiz et al., 2022; Wang et al., 2017).

Furthermore, the presence of numerous mitochondria and antioxidant enzymes in the liver confirms that the liver is the organ where ACR is metabolised, making it particularly sensitive to structural and functional damage (Hong et al., 2019). It is due to the fact that this compound increases oxidative stress, particularly in cells with a high mitochondrial density, because it directly depletes GSH through Michael's addition (Huchthausen et al., 2023). In addition, ACR increases mtROS, lowers mitochondrial membrane potential, inhibits mitochondrial respiration, and disrupts the respiratory chain complexes (Yang et al., 2021). Additionally, studies conducted on human HepG2 liver cells confirm the toxic effects of ACR which caused oxidative DNA damage and consequent loss of the GSH pool (Jiang et al., 2007).

**Fig. 1:**
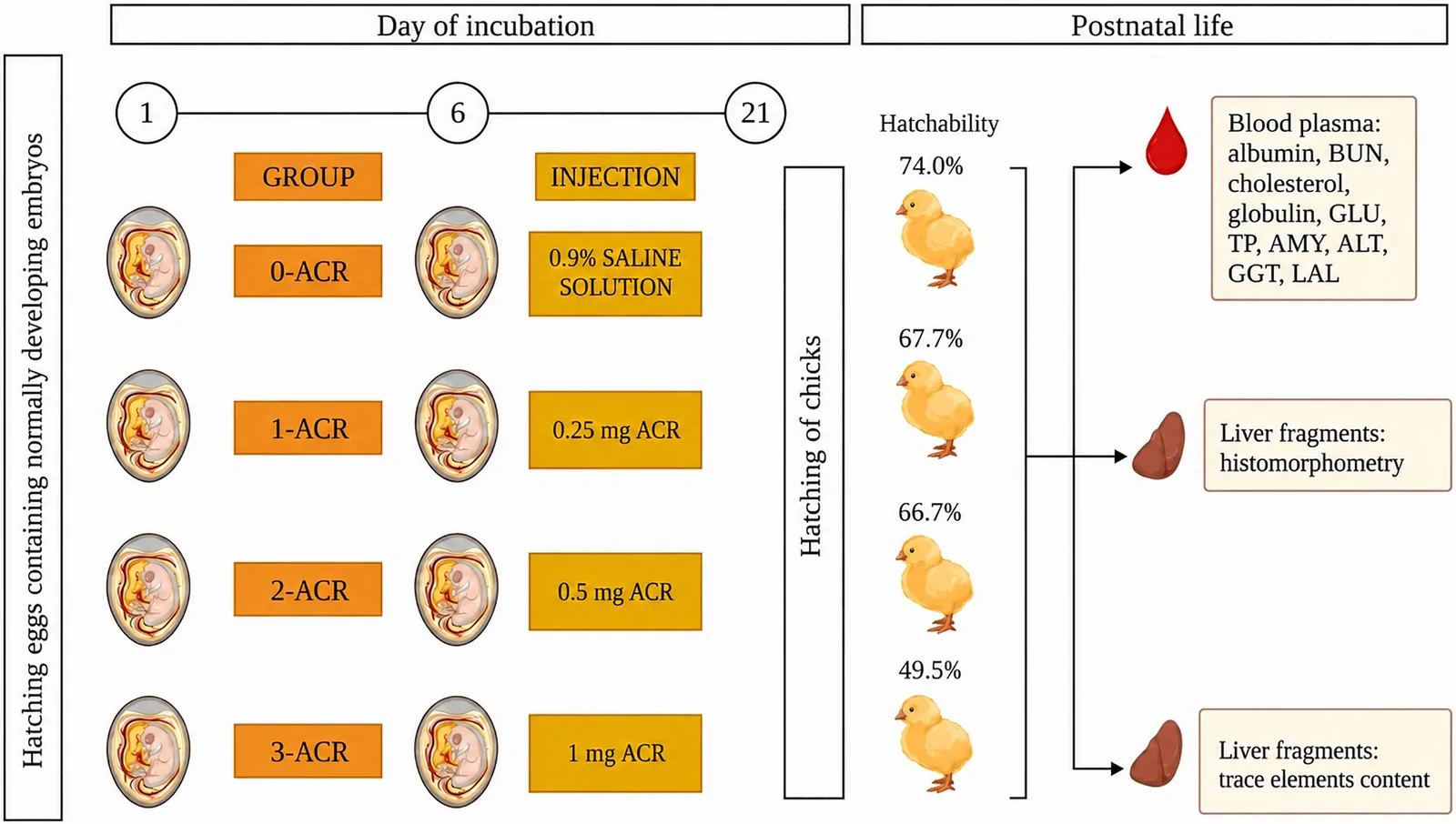
Experimental design scheme (excluding dead embryos from the 4-ACR and 5-ACR groups). Embryos not exposed to the ACR (0-ACR group) and ACR-exposed embryos (administered on day 6): 1-ACR group at a dose of 0.25 mg/egg, 2-ACR group at a dose of 0.5 mg/egg, 3-ACR group at a dose of 1 mg/egg. On day 1 of postnatal life, the chicks were decapitated and blood and liver samples were collected from each group (control and experimental, n = 40) for further analyses. Figure created using BioRender.com.

Previous studies in mammals (mainly rodents) indicate toxic effects of ACR reflected in liver structure as well as biochemical blood tests in adults and their offspring (Marković Filipović et al., 2022; Raju et al., 2015; Tomaszewska et al., 2022). However, such experiments have not been practically carried out on birds, especially on the in ovo model and therefore there is a research deficiency in this area. There are only a few studies that have assessed brain development, oxidative stress, and early neural tube development following embryonic exposure of embryos to ACR, and these focus only on neurotoxicity and oxidative markers (Batoryna et al., 2018; Becit-Kizilkaya et al., 2024). In addition, only a few experiments have examined heavy metal concentrations (as trace elements physiologically present in the body) after ACR poisoning, but their levels were only measured in the blood (Huyut and Arihan, 2018; Yerlikaya and Yener, 2013). Due to the fact that the embryonic liver is in a period of maximal cell proliferation, and is therefore characterized by increased sensitivity to xenobiotics, the current study was planned to evaluate the hepatic structural changes, blood biochemical parameters and trace element levels in the liver of one-day-old chicks that were in ovo administered ACR at different doses (Fig. 1). This experiment will allow us to assess microstructural changes in the liver, which is responsible for detoxifying the body of toxic substances, as well as changes in the levels of the organism's homeostasis indicators in the blood.

## Materials and methods

### Ethical approval

Experiments were conducted according to the guidelines approved by the 2nd Local Ethics Committee for Animal Experiments at the Institute of Pharmacology of the Polish Academy of Sciences in Cracow, Poland (resolution no. 77/2025, of 24 April 2025).

### Animals and experimental groups

The hatching eggs (n = 600) were obtained from a parental flock of Ross 308 chicken broiler (Aviagen) at the Gołaczewy Poultry Farm in Wolbrom (Poland) and transferred to the Department of Zoology and Animal Welfare laboratory at the University of Agriculture in Krakow. All eggs were disinfected with ozone and set in the laboratory incubators (Iglotech, Cracow, Poland). During the setting period (i.e. days 1-18 of incubation, E1-E18), the eggs were incubated at a temperature of 37.8 ± 0.1°C and relative humidity (**RH**) of 50% ± 1%. The setting trays were tilted at a 45° angle and rotated 90° in a one-hour cycle. On the day 19 of incubation (E19), the eggs were transferred to hatching baskets after which the hatching process was carried out at temperature 37.2 ± 0.1°C and RH 55–70% (Fig. 1).

On the 6th day of incubation, the eggs were candled to confirm proper embryo development and to identify and remove unfertilised eggs and dead embryos. The remaining embryonated eggs (n = 576) were divided into six equal groups (n = 96). Next, the eggshell surface above the big (blunt) end of the egg was disinfected with 70% ethanol (Chempur, Poland) and a hole was made by carefully pierce the shell into the air chamber using an 18 G needle (1.20 mm × 40 mm). The solution (100 µL) of ACR (A8887, Merck KGaA, Darmstadt, Germany) was in ovo injected into the allantois using a G20 needle (0.9 mm × 40 mm) with an insulin syringe (1 mL) (Bojarski et al., 2022; Lis et al., 2011), according to the above scheme.

− 0-ACR – control group, injected with 0.9% saline solution (Polfa, Cracow, Poland), which was the solvent for ACR in the other groups
− 1-ACR – experimental group, injected with a solution containing ACR at dose of 0.25 mg/egg
− 2-ACR – experimental group, injected with a solution containing ACR at dose of 0.5 mg/egg
− 3-ACR – experimental group, injected with a solution containing ACR at dose of 1 mg/egg.
− 4-ACR – experimental group, injected with a solution containing ACR at dose of 2 mg/egg
− 5-ACR – experimental group, injected with a solution containing ACR at dose of 4 mg/egg.

After manipulation, the holes were sealed with melted paraffin, and the incubation of eggs was continued. The eggs were re-candled to remove dead embryos on the 10th (E10) and 19th (E19).

The ACR dose and solution volume were based on previous studies (Batoryna et al., 2018; Bojarski et al., 2022; Lis et al., 2011). The injection was performed on the 6th day of incubation (E6) for the following reasons:

1) the hepatic primordium has already formed, but the hepatoblasts are still in an early proliferative state. The embryo is entering the rapid phase of liver growth and vascular development, which occurs between E6 and E9 (Nakayama et al., 2006)
2) egg candling at this stage allows for the confirmation of proper embryo development, as well as the identification and removal of unfertilised eggs and dead embryos
3) the chick embryo has become sufficiently resistant to manipulation (Bruggeman et al., 2003).

At the end of the experiment, after 21 days of incubation the hatchability and quality of chicks were evaluated. Next, ten individuals from each of the groups (40 birds in total) were randomly selected and euthanized by decapitation. The remaining healthy chickens were placed up for adoption. All removed and unhatched eggs were broken open and analyzed.

### Sampling

Immediately after hatching, blood (1-1.5 ml) from 10 randomly selected chicks from each group was collected after decapitation from the jugular vein into heparin tubes and were centrifuged at 3000 × g for 10 minutes at 4°C. Individual plasma samples were kept at −80°C until biochemical analyses (the specimens was not pooled). Liver samples taken in the right lobe from 10 randomly selected chicks from each group were cut for histological investigation and fixed in 10% buffered neutral formalin, while liver samples taken in the left lobe from 10 randomly selected chicks from each group were cut snap frozen in liquid nitrogen and stored at −80°C until subsequent trace elements analyses.

### Blood measurements

Blood plasma concentration of albumin, blood urea nitrogen (**BUN**), cholesterol, globulin, glucose (**GLU**), total protein (**TP**) and activity of amylase (**AMY**), alanine aminotransferase (**ALT**), gamma-glutamyl transferase (**GGT**) and lysosomal acid lipase (**LAL**) were measured using a Catalyst One chemistry analyzer (IDEXX Laboratories, Westbrook, ME, USA) with manufacturer-supplied dry chemistry slides (Chem17 CLIP slide, Idexx Laboratories, USA). This system does not provide chicken-specific analytical calibration of the slides. Therefore, the obtained values were used for comparisons among experimental groups analyzed under identical conditions, i.e. using the same sample matrix, instrument, and analytical procedure and were not interpreted using mammalian diagnostic reference intervals (Brandon et al. 2025). Lithium-heparin plasma was separated by centrifugation and carefully transferred without aspirating the cellular layer.

### Histomorphometric analysis

Fragments from the right liver lobe from 10 one-day-old chicks per group (40 fragments in total) were fixed in 10% buffered neutral formalin for 24 h, washed for 12 h in running water, dehydrated in graded series of ethanol and embedded in paraplast. Prepared paraffin blocks were cut on a Leica Histocore Multicut microtome (LeicaMicrosystems, Nussloch, Germany) at 4 µm thickness sections and placed on 3-aminopropyltriethoxysilane coated microscopic slides. Goldner trichrome staining was used to assess the basic histomorphometric parameters of the liver: total cell number/mm^2^, total hepatocyte number/mm^2^, other cells/mm^2^ (non-hepatocytes cells), total hepatocyte nuclei number/mm^2^, mononuclear hepatocyte number/mm^2^, binuclear hepatocyte number/mm^2^, collagen area, average size of nuclei, perimeter of nuclei, Feret diameter of nuclei, circularity of nuclei (Kiefer et al., 2016; Klebaniuk et al., 2018; Tomaszewska et al., 2015, 2022). Furthermore, this staining allowed microscopic observation and evaluation of liver-specific structures such as portal triads, terminal hepatic venules and lobular and hepatocyte microarchitecture. Histological analysis of the stained liver sections was performed using an Olympus CX43 microscope (Olympus, Tokyo, Japan) and ImageJ software (NIH, Bethesda, USA).

### Determination of metal concentrations

Liver samples weighing approximately 0.2 g to the accuracy of 0.001 g were mineralized using HClO_4_ and HNO_3_ at a ratio of 1:3 for 24 hours. In the next level, the experimental material was subjected to thermal mineralization using a Velp 20/26 mineralizer (Velp Scientifica, Usmate Velate, Italy), where the temperature was increased from 100 to 180°C over 6-7 hours. The content of trace elements, i.e. iron (**Fe**), copper (**Cu**), zinc (**Zn**), nickel (**Ni**), lead (**Pb**) and cadmium (**Cd**) in liver was determined by atomic absorption spectrometry on a Unicam 929 machine (Unicam Ltd., Cambridge, England). Metal content was expressed in mg per kg of wet tissue weight (Drąg-Kozak et al., 2021; Pałka et al., 2022).

### Statistical analysis

Results are expressed as means with standard error (**SE**). Statistica 13.3 software package (TIBCO Software Inc., Palo Alto, USA) was used to conduct one-way analysis of variance (ANOVA) with two-tailed Dunnett’s post-hoc test to compare ACR groups with the control group. Before performing the one-way analysis of variance, the normal distribution was checked using the Shapiro-Wilk test and the homogeneity of variance was examined using the Brown-Forsythe test for all analyzed variables. In the case of a non-normal distribution and/or unequal variance of the data, a logarithmic transformation was applied. For data that did not meet these assumptions, a non-parametric analysis Kruskal-Wallis test with Dunn’s multiple comparisons test was used. A p value of <0.05 was considered statistically significant for all tests.

## Results

### Hatchability

Hatchability was 74.0% in 0-ACR, 67.7% in 1-ACR, 66.7% in 2-ACR and 49.5% in 3-ACR, while all embryos died in 4-ACR and 5-ACR. The majority of 5-ACR embryos (98%) died within 24 h after treatment (E6–E7), while the peak of mortality in the other groups occurred during hatching (E18–E21).

### Blood plasma biochemical analysis

**Fig. 2:**
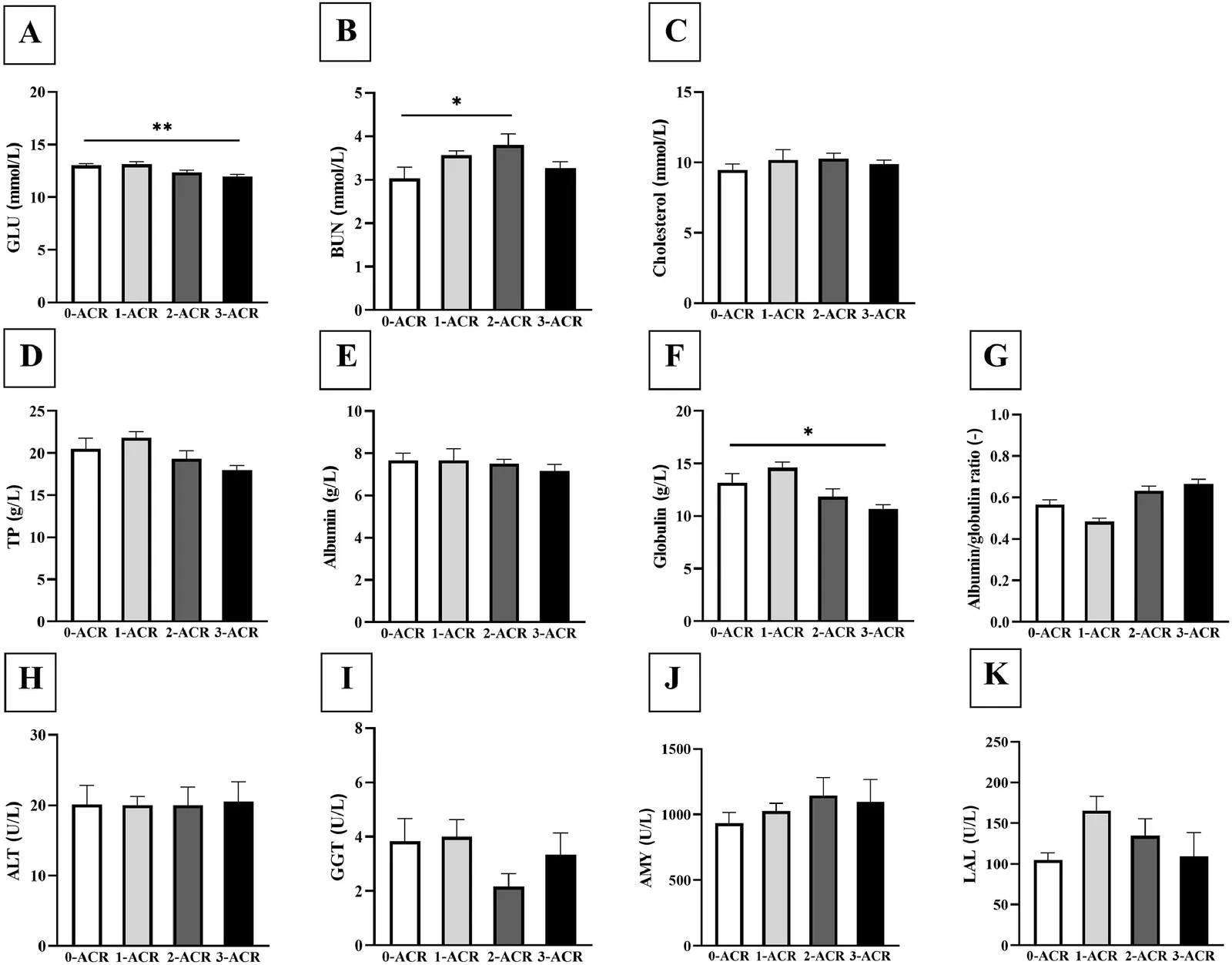
The effect of ACR administration at different doses to chicken embryos on plasma biochemical analysis of hatched chicks. Blood plasma concentration of glucose (GLU) (A), blood urea nitrogen (BUN) (B), cholesterol (C), total protein (TP) (D), albumin (E), globulin (F) and albumin/globulin ratio (G), activity of alanine aminotransferase (ALT) (H), gamma-glutamyl transferase (GGT) (I), amylase (AMY) (J) and lysosomal acid lipase (LAL) (K). Data are presented as mean and standard error. * - indicates differences with p < 0.05; ** - indicates differences with p < 0.01, *** - indicates differences with p < 0.001 (two-tailed Dunnett’s test or Kruskal-Wallis test).

Blood plasma biochemical parameters of one-day-old chicks treated with different doses of ACR are shown in Fig. 2A–K. The GLU concentration was significantly decreased in the 3-ACR compared to the 0-ACR group (P = 0.003) (Fig. 2A). Similarly, globulin concentration was significantly lower in the 3-ACR group compared to the control group 0-ACR (P = 0.041) (Fig. 2F). The concentration of BUN was higher in chicks from the 2-ACR group compared to that from chicks in the 0-ACR group (P = 0.034) (Fig. 2B). No changes in cholesterol, TP, albumin concentrations, albumin/globulin ratio, activity ALT, GGT, AMY and LAL were observed (Fig. 2C–E; G–K).

### Liver trace elements content analysis

**Fig. 3:**
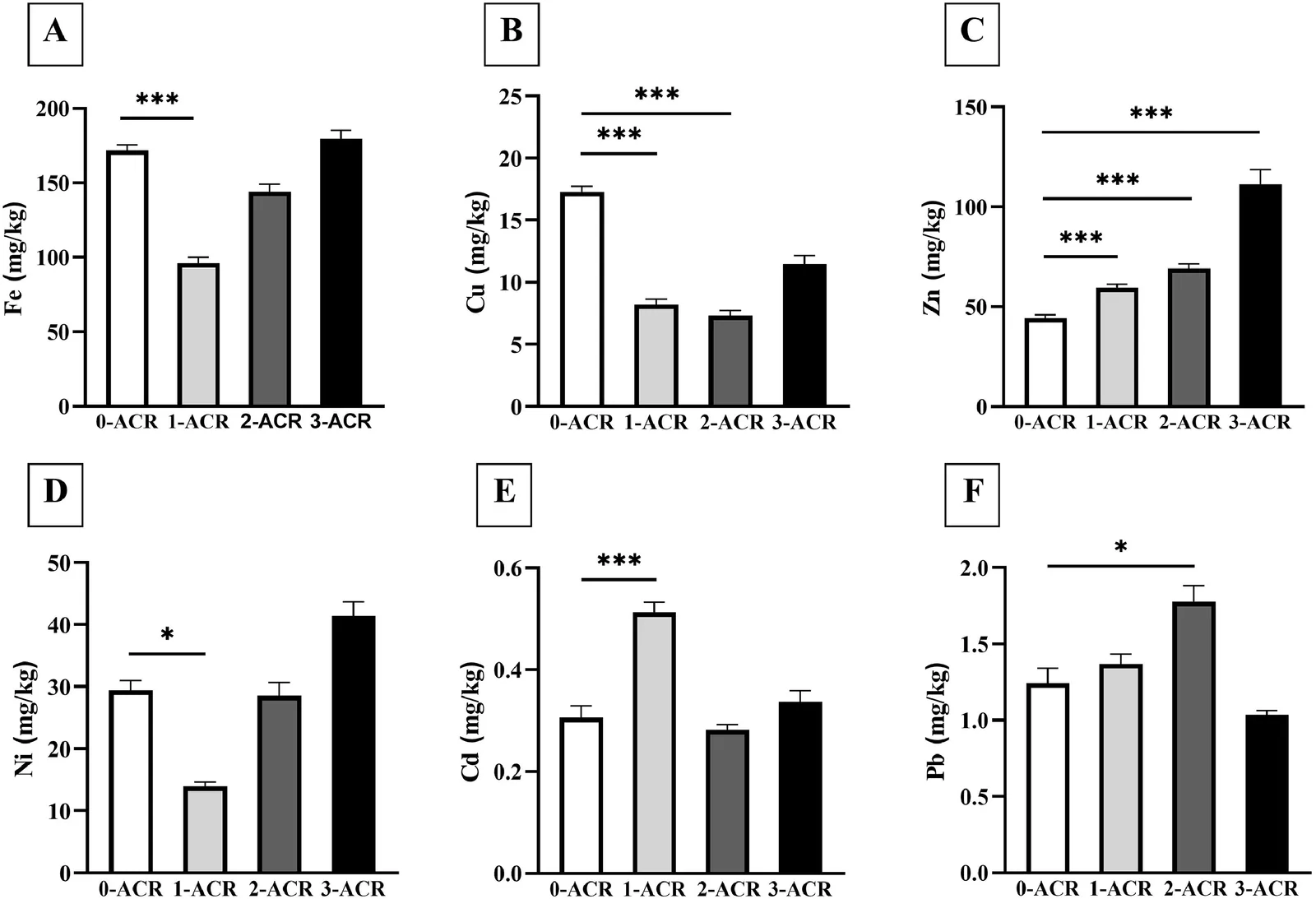
The effect of ACR administration at different doses to chicken embryos on the content of selected trace elements in the liver of hatched chicks. Iron content (Fe) (A), copper content (Cu) (B), zinc content (Zn) (C), nickel content (Ni) (D), cadmium content (Cd) (E) and lead content (Pb) (F). Data are presented as mean and standard error. * - indicates differences with p < 0.05; ** - indicates differences with p < 0.01, *** - indicates differences with p < 0.001 (two-tailed Dunnett’s test or Kruskal-Wallis test).

Fig. 3A–F shows the results of the analysis of the content of selected metals in the liver of one-day-old chicks treated with different doses of ACR. These studies showed a decrease in Fe and Ni content in the 1-ACR animals compared to the 0-ACR control group (P < 0.001, and P = 0.012, respectively) (Fig. 3A and D). Similar interactions were observed in the case of Cu, where significantly lower content of this element was found in the liver of birds from 1-ACR and 2-ACR groups compared to the 0-ACR group (P < 0.001, and P < 0.001, respectively) (Fig. 3B). Notably, the liver of 1-ACR individuals had higher Cd content compared to the liver of 0-ACR individuals (P < 0.001) (Fig. 3E). However, the Pb content was significantly higher in the 2-ACR group compared to the 0-ACR control group (P = 0.013) (Fig. 3F). In addition, the livers of all birds in the experimental groups (1-ACR, 2-ACR and 3-ACR) had higher Zn content compared to the livers of the 0-ACR animals (P < 0.001, P < 0.001, and P < 0.001, respectively) (Fig. 3C).

### Liver histomorphometric analysis

**Fig. 4:**
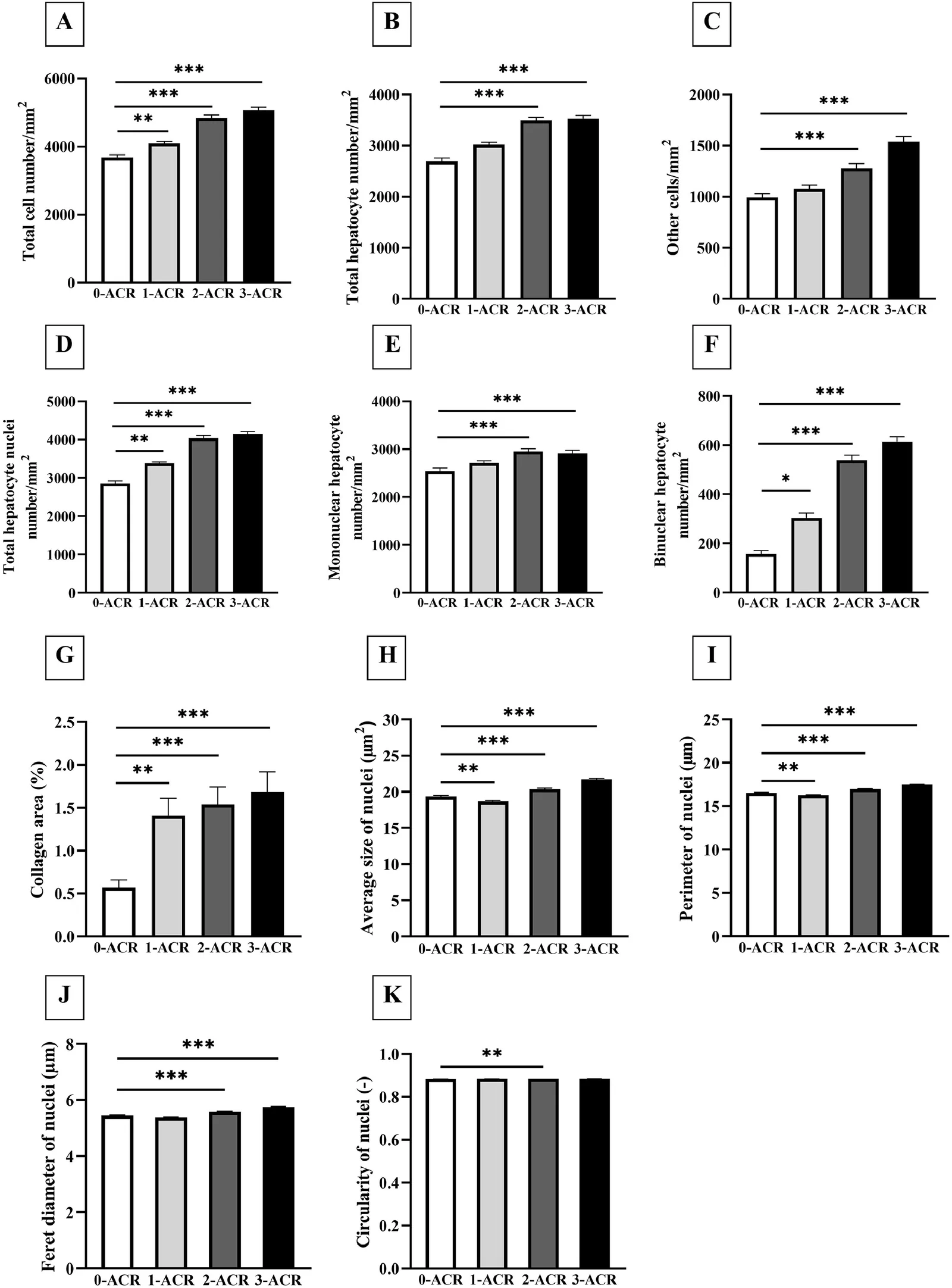
The effect of ACR administration at different doses to chicken embryos on liver histomorphometric analysis of hatched chicks. Total cell number/mm2 (A), total hepatocyte number/mm2 (B), other cells/mm2 (C), total hepatocyte nuclei number/mm2 (D), mononuclear hepatocyte number/mm2 (E), binuclear hepatocyte number/mm2 (F), collagen area (G), average size of nuclei (H), perimeter of nuclei (I), Feret diameter of nuclei (J) and circularity of nuclei (K). Data are presented as mean and standard error. * - indicates differences with p < 0.05; ** - indicates differences with p < 0.01, *** - indicates differences with p < 0.001 (two-tailed Dunnett’s test or Kruskal-Wallis test).

**Fig. 5:**
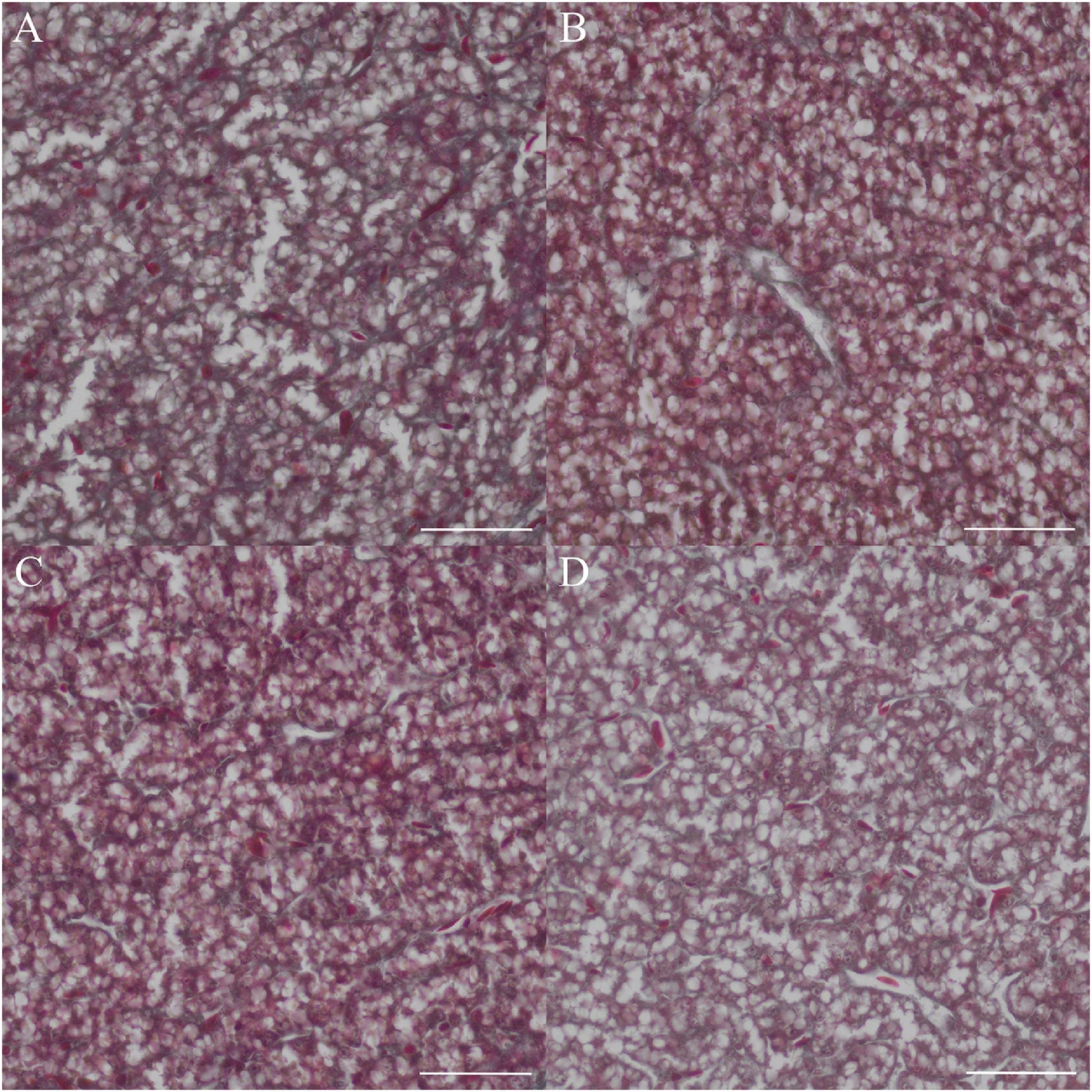
Representative microphotographs of the liver stained using Goldner's trichrome method, obtained from one-day-old chicks treated during the embryonic period with 0.9% saline solution (A) and acrylamide in doses of: 0.25 mg/egg (B), 0.5 mg/egg (C), and 1 mg/egg (D). All scale bars represent 50 μm.

The results of the assessment of histomorphometric parameters of the liver (performed on stained preparations using the Goldner method - Fig. 5) of hatched chicks injected with various doses of ACR during embryonic development, are presented in Fig. 4. Notably, the livers of chicks from all experimental groups had higher total cell number/mm^2^ (P = 0.001 in the case of 1-ACR, P < 0.001 in the case of 2-ACR and 3-ACR), total hepatocyte nuclei number/mm^2^ (P = 0.009 in the case of 1-ACR, P < 0.001 in the case of 2-ACR and 3-ACR), collagen area (P = 0.005 in the case of 1-ACR, P < 0.001 in the case of 2-ACR and 3-ACR) and binuclear hepatocyte number/mm^2^ (P = 0.020 in the case of 1-ACR, P < 0.001 in the case of 2-ACR and 3-ACR) compared to animals from the control group (Fig. 4A, D, G and F). Furthermore, individuals from groups treated with doses of 0.5 mg/egg and 1 mg/egg ACR had a higher total hepatocyte number/mm^2^ (P < 0.001, and P < 0.001, respectively) (Fig. 4B), other cells/mm^2^ (P < 0.001, and P < 0.001, respectively) (Fig. 4C), mononuclear hepatocyte number/mm^2^ (P < 0.001, and P = 0.001, respectively) (Fig. 4E), average size of nuclei (P < 0.001, and P < 0.001, respectively) (Fig. 4H), perimeter of nuclei (P < 0.001, and P < 0.001, respectively) (Fig. 4I) and Feret diameter of nuclei (P < 0.001, and P < 0.001, respectively) (Fig. 4J) compared to the group injected with saline solution. Only the 2-ACR group had higher circularity of nuclei (P = 0.005) (Fig. 4K) than the 0-ACR group. Opposite relationships, a decrease in average size of nuclei (Fig. 4H) and perimeter of nuclei (Fig. 4I) were observed only in the 1-ACR group compared to the 0-ACR group (P = 0.004, and P = 0.006).

## Discussion

The development of the modern animal breeding environment is associated with the exposure of animals to many anthropogenic chemicals. Among them are many xenobiotics such as ACR, as well as certain heavy metals like Cd and Pb. What is important is that many of these substances show strong toxic effects on living organisms (Naiel et al., 2023; Pandey and Madhuri, 2014). The transfer of harmful chemicals to organisms can take place through a variety of routes, including digestive, inhalation, skin, secretions or eggshell. What is interesting to note is that most of the studies on the toxic effects of ACR on liver structure have been focused mainly on mammals especially rodents (Beland et al., 2013; Hułas-Stasiak et al., 2013; Yousef and El-Demerdash, 2006; Yu et al., 2019). Only limited works have been carried out on birds, while such experiments are lacking on the in ovo model. Therefore, it is of interest to investigate the liver structure of one-day-old chicks under the damaging effects of ACR administered in ovo, as environmental factors are among the most important determinants of organismal development in later life.

At the first, it should be noted that in our study, the complete mortality at 2 and 4 mg/egg ACR, with 98% of embryos dying within 24 h at 4 mg/egg, indicates marked acute embryolethality at the highest exposures. However, hatchability observed at 0.25–1.0 mg/egg does support a simple linear dose–response relationship. The predominance of mortality during days 18–21 (E18–E21) at lower doses may reflect sublethal developmental injury that became critical during the energy-demanding hatching process, consistent with evidence that ACR suppresses cell proliferation and promotes apoptosis in chick embryos and that insufficient nutrient availability compromises survival during hatching (Becit-Kizilkaya et al., 2024; Molenaar et al., 2010). Therefore, in-depth analyses of the functioning of individual organs and body systems should help explain the influence of ACR on chicken embryonic development.

Chicken hepatic specification begins in the ventral foregut endoderm at Hamburger–Hamilton stages HH8–9 (Hamburger and Hamilton, 1992). E6 corresponds to approximately HH28–29. By this point, the hepatic primordium has already formed, but the liver is still in an active phase of organogenesis. Chick hepatoblasts express Id3, a regulator associated with proliferation and maintenance in an immature state, from HH12 to HH29. Id3 is no longer expressed in hepatocytes from around HH34 onwards, and its expression pattern is complementary to that of albumin, which is a marker of more differentiated hepatocytes. Thus, E6 marks the end of the early proliferative hepatoblast phase, preceding the later stages of hepatocyte maturation (Nakayama et al., 2006). Furthermore, quantitative three-dimensional analyses of chicken liver development have revealed that the period from E6 to E9 (corresponding to approximately HH29–HH35) is a critical interval for rapid hepatic growth. During this period, the liver increases disproportionately in relation to overall embryo growth, undergoing pronounced expansion of the parenchyma and development of the vascular architecture (Rzhepakovsky et al., 2025). Therefore, acrylamide exposure initiated at E6 occurs at the beginning of a major phase of hepatic expansion and remodelling, after initial liver specification but before later hepatocyte maturation.

ACR has the ability to disrupt endocrine metabolism by impairing endogenous hormonal activity (Matoso et al., 2019). And it is worth remembering that the liver is a very important organ responsible for the production of many blood plasma proteins (albumin, globulins, fibrinogen, transferrin or heparin). Therefore, an ideal analytical material for research is so-called ‘whole’ blood, which has all morphotic and fluid elements as well as serum and plasma. For haematological studies, whole blood is most commonly used, while plasma or serum is used for biochemical studies. Thus, in studies where liver changes were investigated, blood biochemical parameters such as albumin, globulin, cholesterol, GLU, TP, ALP, AMY were most commonly determined (Raju et al., 2015; Tomaszewska et al., 2017, 2021). They can provide useful information on whether and to what extent the function of this organ has been impaired.

One of the most important functions of the liver is its participation in protein metabolism. Thus, it is worth mentioning that albumin is mainly synthesised in hepatocytes and is mainly responsible for binding water. Due to the binding of large amounts of water, oncotic pressure is exerted on the capillary walls. Small-molecule compounds, i.e. hormones, are deposited on albumin molecules and thus provide a specific vector for these compounds in the blood (Sharma and Shukla, 2011). So abnormal levels of this protein may indicate liver damage. However, the available literature lacks data on the effect of in ovo administration of ACR on albumin concentrations. In comparison, there are many studies on the effect of administration of this xenobiotic (in the diet, in water, by gavage) in rodents (Belhadj Benziane et al., 2019; Raju et al., 2015). In addition, there are works on the effects of prenatal programming following ACR administration on the liver structure of rats (Tomaszewska et al., 2022). In the present study, administration of ACR in ovo at different doses did not affect plasma albumin levels in one-day-old chicks, which suggests that this compound does not lead to leakage of protein reserves from hepatocytes. Similar observations were reported by Raju et al. (2016) in 30-week-old F344 rats administered ACR at various doses in the diet. In contrast, several studies have reported that ACR exposure impairs albumin levels, resulting in decreased serum albumin concentrations in rodents (Khudiar and Hussein, 2017; Rivadeneyra-Domínguez et al., 2018). Reduced concentrations of this biomarker may indicate liver damage (Tomaszewska et al., 2022). However, a number of studies have found the opposite effect, specifically an increase in blood albumin levels following exposure to ACR. It is worth noting that these changes were observed following the application of a high dose of the toxicant (Ghorbel et al., 2017; Pazarcı, 2022; Raju et al., 2015; Tomaszewska et al., 2022). In addition, the fact that albumin levels remained unchanged in response to various doses of ACR in our study does not allow us to conclusively state that these doses were insufficient to induce a toxic effect, since, as research indicates, ACR toxicity may be present without changes in albumin levels and may manifest as oxidative stress or histological changes (Rivadeneyra-Domínguez et al., 2018).

Other important proteins produced in the liver are globulins. Plasma globulins include carrier proteins or immunoglobulins, but the latter are only synthesised in lymphoid tissue. Hence, most of the non-immunoglobulin globulins in serum are synthesized and stored precisely in the liver. Significantly, changes in the levels of this protein are observed in the progression of many metabolic liver diseases (Fallatah and Akbar, 2010; Liu et al., 2015). Thus, there is also much evidence that exposure to ACR in high doses leads to a reduction in globulin concentration and consequent liver damage (Khudiar and Hussein, 2017; Yousef and El-Demerdash, 2006). This is also confirmed by the results obtained in the present study, where a significant reduction in plasma globulin levels was demonstrated in the 3-ACR group (with the highest dose of ACR used) compared to the 0-ACR control group. This reduction in liver protein levels may be due to delayed protein synthesis or changes in protein metabolism (Sharma et al., 2008). However, the albumin/globulin ratio is also an important indicator for assessing overall health. Because if albumin is not produced properly in many liver diseases then the ratio of albumin to globulin will be low. What is important in the present study is that this parameter did not differ significantly between the experimental and control groups.

The varying results do not relate to the parameters described earlier, but also to TP. Thus, an increase in blood TP values in animals exposed to ACR was observed by Ghorbel et al. (2017) and Tomaszewska et al. (2022). In contrast, experiments conducted by Raju et al. (2015), Rivadeneyra-Domínguez et al. (2018) and Belhadj Benziane et al. (2019) show different results, i.e., reductions in TP concentrations in individuals exposed to the experimental agent compared to the control group. It is interesting to note that our study shows that ACR administration has no effect on the value of this parameter. These results confirm earlier observations by Martiniakova et al. (2020).

Another important trait that can indicate organ failure is blood cholesterol levels. Also significant, cholesterol can be synthesized in the liver or absorbed from the diet. It has many important functions in the body, including being a precursor of steroid hormones and is necessary for the synthesis of fatty acids. Cholesterol is metabolized in the liver and excreted with the bile. Thus, some studies find higher serum cholesterol levels in individuals exposed to ACR compared to a control group (Mahmood et al., 2016). On the other hand, there are some works where no effect of ACR was observed on the value of this parameter, which was also noted in our study (Pazarcı, 2022; Raju et al., 2015, 2016). However, low blood cholesterol is associated with liver dysfunction (Jiang et al., 2014). Therefore, changes in plasma lipoprotein concentrations are a sensitive and simple marker for assessing metabolic disorders of the liver.

An important indicator of good body condition is the measurement of GLU in the blood, which reflects the hormonal balance of individuals. The proper concentration of GLU in the blood depends on the rate of release of this sugar by the liver or intestines, as well as the rate of uptake by other tissues. It is worth mentioning that the accumulation of GLU in the form of glycogen in the liver is necessary for supplying GLU to other tissues that cannot store this sugar. It should be noted that there are no uniform results in the available literature regarding the effects of ACR on GLU metabolism. Raju et al. (2016) and Karimani et al. (2021) observed no effect of ACR on the concentration of this sugar in the blood of rats. Some authors observed increase level of GLU (Ghorbel et al., 2017; Tomaszewska et al., 2022; Yue et al., 2020) or its decrease (Rivadeneyra-Domínguez et al., 2018). A similar decrease was observed in the present study in a group of animals given ACR at a dose of 1 mg/egg compared to individuals given 0.9% saline solution. What is important is that numerous studies indicate that many xenobiotics induce the constitutive androstane receptor (**CAR**) in hepatocytes, leading to increased expression of cytochrome P450 2B6 (**CYP2B6**) and decreased expression of phosphoenolpyruvate carboxykinase (**PEPCK**) and glucose-6-phosphatase (**G6Pase**), and, as a result, inhibits hepatic gluconeogenesis, reduces hepatic glucose production, and improves insulin sensitivity (De Battistis et al., 2025; Küblbeck et al., 2020; Wang et al., 2023). However, there are no data from animal models, including mammals and birds, confirming the activation of such a mechanism following ACR intoxication. On the other hand, many exogenous compounds (including ACR) lead to hyperglycemia, increase gluconeogenesis and glycogenolysis, and inhibit glycolysis (Guo et al., 2026; Yue et al., 2020). Thus, the observed decrease in GLU levels in birds treated with a high dose of ACR in our experiment may be related to impaired utilization of nutrients from the yolk sac, since, as studies confirm, this is a site exposed to xenobiotics, which may metabolize it (Mosallanejad et al., 2022; Wong and Uni, 2021). Furthermore, this may indicate that chicks treated with high doses of ACR may experience impaired activity of enzymes (for example, glycogen synthase 1 – **GYS1**) responsible for glycogen accumulation, abnormal glycogen storage, and consequently liver dysfunction. Interestingly, a decrease in GLU levels has been observed in certain metabolic liver diseases in mammals, such as cirrhosis (Ulicna et al., 2003).

Measurement of the amount of BUN is used to assess endocrine disorders, which is the end product of hepatic detoxification of ammonia. A significant reduction in this parameter following ACR induction was found in an earlier study by Raju et al. (2015) conducted on rats. The opposite effect was obtained by Rivadeneyra-Domínguez et al. (2018) where a significant increase in BUN levels was observed in a group of individuals induced with a dose of 50 mg/kg ACR compared to the control group. Also in the present study, a significant increase in the value of this parameter was observed, but only in the 2-ACR group (0.5 mg/egg ACR) compared to the 0-ACR group (0.9% saline solution).

ALT testing is one of the primary methods for diagnosing liver damage. ALT is one of the liver enzymes used as a marker of liver cell damage. Many studies on mammals have shown that exposure to ACR results in an increase in ALT levels, which leads to hepatocyte damage (Al Syaad et al., 2023; Ghorbel et al., 2017; Karimani et al., 2021; Rivadeneyra-Domínguez et al., 2018; Singh et al., 2015). Furthermore, intermediate metabolites produced during the biotransformation of ACR, such as glycidamide, may induce hepatotoxicity and also induce an increase in liver enzyme activity in blood plasma. In contrast, other experiments conducted on rats did not indicate a negative effect of ACR on the value of this parameter (Kovac et al., 2015; Raju et al., 2016), which is consistent with the results of the present study.

Another test that is helpful in the diagnosis of liver disease is the measurement of AMY levels. AMY is an enzyme from the hydrolase class that is responsible for the initial digestion of carbohydrates. In the available literature, there is little data on the effect of ACR on the value of this parameter. Raju et al. (2015) and Raju et al. (2016) showed no significant effect of ACR on serum AMY activity in male F344 rats. Similar results were obtained in our work, where we demonstrated no effect of the administered substance on the activity of this enzyme. Other enzymes found in the liver are GGT and LAL. Interestingly, these are important indicators used in the diagnosis of liver disease in humans (Pezzilli, 1999; Xing et al., 2022). Unfortunately, such information is lacking for studies on the effects of ACR on these biochemical parameters in animal blood. Hence, in the present study, the effect of ACR on LAL and GGT activity in the plasma of one-day-old chicks was investigated for the first time. The result showed no significant differences between the control and experimental groups for both LAL and GGT levels. Furthermore, it should be emphasised that there are some limitations to the interpretation of our results. To date, there are no defined physiological standards for poultry and thus chicks for albumin, globulins, cholesterol, GLU, BUN, TP, ALT, GGT, AMY, LAL. Nevertheless, on the basis of the results obtained in our study, it can be concluded that high doses of ACR affect the levels of certain biochemical blood parameters, i.e. GLU, BUN, globulins of one-day-old chicks, which may lead to changes in liver metabolism.

Important roles in liver metabolism and function are also played by micronutrients such as Fe or Zn (Ullah et al., 2023). These minerals are essential for many biological functions related to cell growth and development (Wessels et al., 2017). The study confirms the possibility of forming ACR complexes with metals due to the presence of an amide group in peptides and proteins (Girma et al., 2006). Therefore, we decided to investigate the content of certain elements, i.e. Fe, Cu, Zn, Ni, Cd and Pb, in the liver of one-day-old chicks exposed to ACR.

As is well known, Cu is an important component of the liver, as well as bones, muscles, brain or kidneys (Fu et al., 2025). Thus, the liver is the main storehouse of Cu and allows it to be stored and participate in metabolic processes. In biological systems, it occurs in the form of Cu^+^ and Cu^2+^ ions. However, in cells it does not occur in ionic form but is bound to low molecular weight proteins (Collins, 2021; Roberts and Sarkar, 2008). The role of this element in physiological processes includes, but is not limited to, the activation of many enzymes such as Cytochrome c oxidase (**COX**), Lysyl oxidase (**LOX**) or Tyrosinase (**TYR**), the synthesis of collagen and elastin, the production of red blood cells through the binding of iron to heme in haemoglobin (Roberts and Sarkar, 2008). Furthermore, Cu is responsible for energy balance, mitochondrial respiration and oxidative stress (Festa and Thiele, 2011). Despite such important biological roles of Cu in physiological processes, studies on the effect of ACR on Cu content in the liver of birds and mammals are still lacking. In contrast, the available literature provides information on the effects of ACR administration on plasma Cu levels in mammals. Huyut and Arihan (2018) did not indicate a significant effect of ACR administration at a dose of 20 mg/kg/day (using a gastric gauge) on rat plasma Cu levels. The present experiment showed that in ovo administration of ACR at a dose of 0.25 mg per egg and 0.5 mg per egg leads to a significant reduction in liver Cu content. As Szymczyk and Zalewski (2002) and Collins (2021) suggest, both Cu deficiency and excess can be dangerous and lead to many pathophysiological changes. The data in the literature show that Cu deficiency can lead to numerous metabolic disorders involving the musculoskeletal, cardiovascular or nervous systems (Fu et al., 2025). Deficiency of Cu in birds results in disorders of growth and thus ossification, changes in feather pigmentation, deformation of eggs and consequently can lead to anemia (Berwanger et al., 2018; Leeson, 2009). The reduction in Cu content in the present study is perhaps related to the formation of a Cu-ACR complex where the oxygen atom of the carbonyl group binds to the metal center (Girma et al., 2006; Reedijk, 1971), which can consequently lead to oxidative stress and thus liver cell damage. It is worth noting that Cu is an important element that supports the metabolism of Fe. As a trace element in the organism, Fe can be contained in metabolically active compounds (enzymes, hemoglobin or myoglobin) as well as organs such as the liver and spleen. In the liver of mammals and birds, Fe is stored in the form of ferritin, which stabilizes the redox status of Fe. Thus, Fe is most often maintained in chelated form in the body when it is in cells or body fluids. Animals have the ability to properly manage Fe for many metabolic purposes with Fe supply coming from intestinal absorption, so the mineral does not accumulate and cause damage (He et al., 2022; Klasing et al., 2012). However, sometimes certain factors can lead to disorders of Fe metabolism. This experiment is the first to examine the effect of in ovo administration of ACR on the content of selected elements, including Fe, in the liver of one-day-old chicks. The result showed a significant decrease in this bioelement under the influence of this toxicant, but only in the 1-ACR group (treated with a dose of 0.25 mg ACR per egg). There were no such relationships for the other groups. In the available literature, there is no information on the effect of ACR administration on liver Fe content. However, there are some studies where blood levels of this component were checked after exposure to ACR. Similar to the present work, a decrease in serum Fe levels in rats exposed to ACR was noted by Yousef et al. (2022). In contrast, Raju et al. (2015) showed no changes in blood Fe levels in rats after dietary exposure to ACR. Similarly, Tomaszewska et al. (2022) observed no difference in the blood Fe levels of young rats whose mothers were exposed to ACR. This is consistent with the results of our experiment in the 2-ACR and 3-ACR groups. It is important to remember that both deficiency and excess of Fe in the body can have their negative consequences. Excess of this element can lead to organ damage and thus impaired function (iron storage disease ISD). Among birds, such a problem can lead to ascites, cardiomegaly, hepatomegaly and even death as a result (Klasing et al., 2012). In turn, the decrease in liver Fe levels observed in our experiment after exposure to ACR at a dose of 0.25 mg/egg may indicate depletion of liver Fe stores in this group, which can lead to disorders such as microcytic anemia (Tomaszewska et al., 2022; Yousef et al., 2022).

Another important trace element in the liver is Zn, which has a wide variety of biological functions. Zn acts as a growth cofactor and an important immunoregulator and cytoprotector, showing antioxidant, anti-inflammatory and anti-apoptotic effects (Zalewski et al., 2005). This is made possible, among other things, by antagonism to metals with redox activity, such as Cu and Fe (Powell, 2000). In addition, this mineral is present in numerous enzymes such as DNA and RNA polymerases, affecting the synthesis of proteins, hormones and red blood cells. Significantly, organs such as the muscles, bones, prostate, liver and kidneys are the main reservoir of Zn in the body and remain in a state of dynamic exchange equilibrium with its fraction present in the blood serum (King et al., 2001). As such, it is an essential mineral for the proper functioning of the liver, which also plays a key role in regulating Zn homeostasis in the body. Therefore, changes in Zn levels can lead to a number of disorders and diseases of this organ. In our study, data from liver elemental composition analysis showed significantly higher Zn values in the 1-ACR, 2-ACR and 3-ACR groups compared to the 0-ACR control group. Unfortunately, as with other trace elements, there is a lack of data on the effects of ACR on their levels among birds, and only a little information is available for mammals. A study conducted on rats by Huyut and Arihan (2018) shows that administration of ACR leads to lower plasma Zn levels. Also, Yerlikaya and Yener (2013) observed a reduction in the level of this element in male rats receiving ACR at a dose of 2 mg/kg and 5 mg/kg compared to the control group. The results of Zn deficiency and excess in the organism of mammals are well known (Henke et al., 2023; Prasad, 2009; Siow, 2018). Cai et al. (2025) indicate that a deficiency of this trace element leads to stunted growth, skeletal deformities, reduced nutrient utilization, and lower production performance in animals, as well as reproductive dysfunction in both females and males. In turn, excess Zn causes adverse changes at the cellular level by inducing oxidative stress, leading to mitochondrial dysfunction and, consequently, cell death (Yang et al., 2023, 2023). Therefore, the increase in Zn levels observed in our work may lead to impaired liver function (Wang et al., 2024). Unfortunately, available literature lacks evidence to confirm that such an increase in Zn levels could result from the organism's compensatory response to oxidative stress. However, this may be an indirect effect of ACR metabolism influencing Zn distribution, leading to disturbances in metal homeostasis.

However, it is known that other trace elements, i.e. Ni, Cd or Pb, are also present in healthy liver, while their content is very low. Thus, Ni has important functions in the organism and is essential for the normal course of erythropoiesis, the functioning of intestinal microflora, and may act as a cofactor in the absorption of iron in the gut (Pieczyńska et al., 2021; Zambelli and Ciurli, 2013). In addition, it influences the metabolism of other trace elements such as Cu, Zn and molybdenum (**Mo**), which has been confirmed in animal studies through observed changes in their distribution in the liver and kidneys (Nielsen, 1991). Previous studies on the ACR-Ni relationship are limited, and to our knowledge there are no studies conducted on poultry especially under in ovo conditions. In the current study, liver Ni content was significantly reduced in a group of birds injected with a dose of 0.25 mg/egg compared to individuals in the control group injected with 0.9% saline solution. Importantly, this mineral has a synergistic effect with Fe by aiding its absorption in the intestines, while a deficiency of Ni can result in lower Fe concentrations (Singh et al., 2021). Such relationships were observed in the present study, where we also found lower Fe levels in the 1-ACR group. A research conducted by Shah et al. (2011) on children showed a reduction in blood Ni concentrations in children with anemia compared to healthy children. Therefore, presumably such correlations observed in birds in our study may indicate the possibility of anemia in chicks exposed to ACR at a dose of 0.25 mg/egg. This effect has never been observed before and should be further investigated with an extension to the analysis of these factors in the blood of birds.

On the other hand, Cd is an element that naturally occurs in healthy liver in small amounts. To date, its biological function is not well understood and it is considered one of the heavy metals toxic to the organism. This mineral is primarily deposited in the liver and kidneys, but also in the lungs, brain or heart (Niture et al., 2021). Importantly, numerous studies indicate that overexposure to Cd is associated with genotoxicity, cytotoxicity and carcinogenicity (Joseph, 2009; Lv et al., 2024; Nagaraju et al., 2022). However, it should be noted that there is a lack of studies on the effects of ACR on Cd levels in various tissues, i.e. blood or liver. Nevertheless, there is work on Cd's interaction with other elements, e.g. Zn (Brzóska and Moniuszko-Jakoniuk, 2001). In our study, we observed a significant increase in Cd as well as Zn content in the liver in the 1-ACR group compared to the 0-ACR group. Possibly the increased Cd content in the liver of birds injected with ACR 0.25 mg/egg consequently leads to Zn disturbances among this group. Notably, many studies to date confirm that Zn counteracts the toxic effects of Cd, and perhaps such a mechanism occurred in this experiment of 2-ACR and 3-ACR groups (El Heni et al., 2011; Pan et al., 2017). Because we did not observe any significant changes in Cd levels in these groups, whereas Zn levels increased significantly compared to the control group. However, the lack of precisely defined Cd-ACR relationships in the organism limits the interpretation of the results on a model like one-day-old chicks.

Similar to Cd, Pb is a toxic heavy metal that accumulates in the liver and its biological function is also not yet understood. Pb has a high degree of toxicity and negatively affects the structure and function of many organs and tissues, such as the liver, kidneys, nervous and reproductive systems (Aldahmash and El-Nagar, 2016; Wani et al., 2015). Although in the present experiment the animals were not given Pb, administration of ACR at a dose of 0.5 mg/egg significantly increased Pb levels in the liver of one-day-old chicks, while the levels of this element in the other experimental groups (1-ACR and 3-ACR) remained similar to those in the control group. These observations are consistent with an earlier study by Huyut and Arihan (2018), where the authors showed a significant increase in plasma Pb levels in ACR-treated Wistar-Albino rats compared to control individuals. Such excessive amounts of this mineral in the liver can cause liver damage by inhibition and activation of enzymes primarily monooxygenase of the group of cytochrome P-450 (Degawa et al., 1996). Some limitation in interpreting the above results is the fact that, as yet, there are no specific physiological standards for trace elements content in the liver of poultry, especially one-day-old individuals. Furthermore, it is worth noting that Cd and Pb in the bodies of unhatched chicks may originate from environmental contaminants transferred by the mother (maternal transfer) or absorbed through the porous eggshell. These heavy metals enter the hen’s digestive system mainly through contaminated soil, water, and commercially available poultry feed (Iqbal et al., 2023).

It is important to remember that ACR is a compound made up of carbon, hydrogen, nitrogen and oxygen atoms and contains a conjugated double bond and an amide group in its structure. The amide functional group allows the formation of hydrogen bonds, which affects the interaction properties of the molecule. ACR can coordinate with transition metal cations via oxygen from the C=O group, nitrogen from the NH₂ group, or both once. Therefore, this chemical can react with a number of chemical compounds through, for example, a vinyl or amide group (Girma et al., 2006). Research in coordination chemistry indicates that ACR forms complexes with transition metal ions, particularly Fe²⁺ and Cu²⁺, most commonly as a monodentate ligand coordinating via the oxygen atom of the carbonyl group (Girma et al., 2006). The formation of ACR complexes with Fe²⁺ and Cu²⁺ ions may temporarily reduce the bioavailability of these ions to transport proteins and metal-dependent enzymes and alter their redox properties (Huyut and Arihan, 2018; Mori et al., 2022). Furthermore, metal complexes with ACR derivatives may exhibit catalytic activity and, in the presence of hydrogen peroxide, participate in reactions leading to the formation of highly reactive hydroxyl radicals, which could potentially exacerbate oxidative stress and lead to cellular damage (Mori et al., 2022). In addition, both free ACR and its metabolites can induce oxidative stress by depleting GSH stores, disrupting the function of the mitochondrial electron transport chain, and increasing the production of reactive oxygen species (**ROS**). Consequently, this may lead to damage to hepatocytes and, thus, impair the function of proteins responsible for the transport and storage of metals, which could potentially lead to a disruption of Fe²⁺ and Cu²⁺ homeostasis (Quasmi et al., 2025). However, as research indicates, the mechanism of this action is not fully understood to date, and despite the apparent interaction between toxic substances such as ACR and bioelements necessary for life, research in this area is still lacking (Huyut and Arihan, 2018). Nevertheless, based on experiments conducted on suckling models, it can be hypothesized that ACR disrupts the distribution of endogenous trace elements.

The liver is one of the most metabolically active organs, playing a key role in processing substances obtained from the absorption of yolk, hematopoiesis, carbohydrate, protein, and fat metabolism, storage of glycogen, vitamins, and iron, as well as detoxification. What is important is that among birds, fat synthesis takes place in this organ, and not in adipose tissue, as is the case in mammals (Bao et al., 2023). Therefore, the liver plays an important role immediately after hatching, providing energy for the rapid growth and development of chicks. The main cells that make up this organ are hepatocytes, but it should be noted that its structure also includes Disse spaces, stellate cells, sinusoids with Kupffer cells and hepatic triads. The liver of one-day-old chicks is relatively large in relation to body weight, has a light color, and hepatocytes exhibit a characteristic foamy morphology resulting from intense accumulation of lipids and glycogen (Davies, 2000). Interestingly, in contrast to mammals, this organ in birds has significantly less connective tissue, which means that it does not have the typical lobular structure and has reduced structural strength (Turk, 1982). Both hepatocytes and sinusoids are surrounded by a sparse cross-linking network of type III collagen fibers. Liver cells include 80% hepatocytes, which are pyramid-shaped and have a distinct, large oval cell nucleus with loose chromatin and a distinct nucleolus (Wong and Cavey, 1992; Zaefarian et al., 2019). Hepatocytes also contain numerous mitochondria with granules, lysosomes, rough and smooth endoplasmic reticulum, Golgi apparatus and other organelles. Importantly, these cells perform the most functions of all the cells in the body (Schulze et al., 2019). Therefore, conducting analyses of the liver microstructure provides valuable information on changes in the morphology of this organ under the influence of ACR. This is the histomorphometric analysis of the liver that provides quantitative information on the microscopic structure of the tissue and enables a precise assessment of the degree of damage, regeneration processes, and pathological changes within it. Some studies have shown that exposure to ACR causes liver cell toxicity (Awad et al., 1998; Marković Filipović et al., 2022). However, these experiments were mainly conducted on mammals in both in vitro and in vivo systems, while analyses on avian models, and thus in ovo models, are still lacking. Significantly, ACR toxicity manifests itself through oxidative stress and the production of ROS, to which the liver is particularly sensitive (Cichoż-Lach, 2014). Studies indicate that ACR inhibits the activity of antioxidant enzymes, and excessive ROS production leads to oxidative stress in the liver and is the main cause of hepatocyte apoptosis activation (Zhang et al., 2023). Consequently, this may cause the degradation of actin filaments and dysregulation of Ca²⁺ homeostasis in hepatocyte mitochondria (Biagioli et al., 2008; Dai et al., 2013). Ali et al. (2020) demonstrated that administering ACR to albino rats at a dose of 20 mg/kg body weight for 6 weeks leads to vacuolization of hepatocyte cytoplasm, congestion of the central vein, and apoptosis of liver cells. Similarly, Marković Filipović et al. (2022) observed changes in the microarchitecture of the liver of rats exposed to ACR at a dose of 50 mg/kg body weight, where animals treated with the toxic substance had significantly lower hepatocyte cytoplasm volume density and significantly higher hepatocyte nucleus volume density compared to the control group. In turn, Belhadj Benziane et al. (2019) in studies conducted on 4-week-old Wistar rats (which were administered ACR for 2 months) found macrophage pigment deposits in the liver, which is probably the result of hepatocyte degeneration, blood stasis in the vessels, and inflammatory mononuclear cell infiltration. These observations are consistent with the results obtained in the studies by Rawi et al. (2012) and Ghorbel et al. (2017), where the authors demonstrate mononuclear inflammatory cell infiltration, blood vessel dilation, and hepatocyte necrosis in the liver of rats treated with ACR. However, Raju et al. (2015) in their experiment conducted on 6-week-old male F344 rats showed mild hyperchromasia and anisokaryosis of hepatocytes in all three groups receiving the toxic substance at doses of 5, 10 and 50 mg/kg of diet. It is worth noting that the above-mentioned studies were conducted on mammals, in which the research procedures were performed at later stages of ontogenetic development and were based on subjective histopathological analyses. To our knowledge, only Tomaszewska et al. (2022) conducted a study on a rat model to investigate the nutritional ACR of maternal programming in the context of changes in liver function and basic hematological and oxidative parameters in weaned pups, where the authors performed quantitative measurements within the liver based on microscopic images. Thus, they demonstrated a reduction in the number of hepatocytes and the total number of cells in the liver of offspring from the medium ACR exposure group compared to individuals from the control group. Furthermore, the authors observed a significant reduction in the number of binucleated hepatocytes with a simultaneous increase in the number of other cells in all groups of offspring whose mothers were treated with ACR compared to offspring from the 0-ACR group. Although the results obtained in this study are somewhat different, in all groups treated with various doses of ACR during the embryonic period, an increase in total cell number/mm^2^, total hepatocyte nuclei number/mm^2^, binuclear hepatocyte number/mm^2^ and collagen area was observed in relation to the control group. In addition, the livers of individuals treated with doses of 0.5 mg/egg and 1 mg/egg also demonstrated increased total hepatocyte number/mm^2^, other cells/mm^2^, mononuclear hepatocyte number/mm^2^, average size of nuclei, perimeter of nuclei and Feret diameter of nuclei compared to those injected with saline solution. The only decrease in the parameters of average size of nuclei and perimeter of nuclei was observed in group 1-ACR compared to group 0-ACR. It is worth mentioning that the liver of newly hatched chicks is characterized by dynamic cell reorganization, and the increase in the number of hepatocytes containing two nuclei in the experimental groups may indicate processes related to regeneration or abnormal cytokinesis (Guidotti et al., 2003). The authors suggest that binuclear hepatocytes are formed as a result of incomplete cytokinesis, in which the nucleus divides but the cytoplasm does not, resulting in two diploid nuclei in a single cell. This type of nuclear differentiation and increased genetic material constitute an adaptive mechanism that strengthens the resistance of cells to stress factors and supports hepatocyte renewal processes and may also indicate the course of liver regeneration after exposure to a toxic agent such as ACR. On the other hand, the occurrence of binucleated hepatocytes may be associated with pathological failure of cytokinesis and serve as a marker of mitotic abnormalities caused by oxidative stress (De Santis Puzzonia et al., 2016). Furthermore, reports by Tomaszewska et al. (2014) suggest that the growth of this type of cell may be associated with increased metabolic activity in the liver. On the other hand, the increase in the number of mononuclear hepatocytes in the livers of individuals treated with high doses of ACR (0.5 mg/egg and 1 mg/egg) in our study may indicate liver regeneration. Additionally, the increase in other cells in these experimental groups suggests that such high doses of the toxic substance induce an increase in the number of phagocytic cells, such as Kupffer cells, within the liver of one-day-old chicks as a result of inflammation. According to available literature data, these cells, once activated, can participate in repair processes within this organ (Tomaszewska et al., 2022). However, the microstructural changes observed in this study are not reflected in the levels of liver enzyme markers (e.g., ALT). As well, histomorphometric measurements within hepatocytes are important biomarkers of the metabolic state of these cells and may be an indicator of altered metabolic activity (Rašković et al., 2016; Tomaszewska et al., 2014). Thus, the changes in hepatocyte nucleus size observed in our experiment in the livers of animals exposed to ACR may indicate changes in non-chromosomal protein content and related adaptive transformations of cellular functions (Ivanović-Matić et al., 2000; Strüssmann and Takashima, 1990). According to Lackner et al. (2008), such changes may indicate ballooning degeneration, which is characterized by generalized swelling of the cell and its significant enlargement. This association is due to the accumulation of intracellular fluid caused by microtubule dysfunction and disturbances in the process of protein secretion. In addition, what can be observed in our work is that the circularity of hepatocyte nuclei responds nonlinearly to ACR, and this may be due to the fact that the shape of the nucleus depends not only on the dose of the toxic substance but also on cytoskeletal mechanics, redox status, and proliferation.

An important component in the structure of the liver is also collagen. Type I, III, and V collagen primarily provide the appropriate structure and are found in the interstitial matrix, while type IV collagen provides a scaffold for polarized cells and is located in the basement membrane (Karsdal et al., 2020). Apart from its structural functions, collagen plays an important role in repair processes and cell signaling. Observations by Perepelyuk et al. (2013) indicate that after liver damage, it is mainly stellate cells that synthesize collagen and induce extracellular matrix remodeling, thereby promoting tissue repair. Furthermore, research by Masuzaki et al. (2021) shows that collagen participates in liver homeostasis through its interaction with integrins. In our study, detailed histological analysis showed a significant increase in collagen content in all experimental groups, which may result from increased production of free radicals induced by ACR and lead to increased organ stiffness and consequently to fibrosis (Chen et al., 2019). This may also be the result of changes in the composition of extracellular matrix (**ECM**) proteins due to organ damage, leading to matrix overproduction and inflammation (Stalnikowitz and Weissbrod, 2003). It can be summarized that modifications in collagen structure as a result of exposure to ACR may induce changes within liver cells, which is important in regulating and mediating the response to organ damage. Nevertheless, it is worth noting that in our study, the basic metabolic markers (cholesterol, TP, albumin) and liver function markers (ALT, GGT) did not change under the influence of the toxic substance, despite structural liver damage confirmed by histological examination and the measurement of selected trace elements. This effect may stem from the fact that the extent of liver damage was insufficient to induce detectable changes in plasma, or that the study was conducted at a stage when histological changes had not yet been followed by a biochemical response. Thus, the structural changes induced by ACR are most likely caused primarily by oxidative stress and the inflammatory response observed in the tissue, and this damage is still in its initial stages.

In summary, this is the first report describing the effects of embryonic exposure to ACR on liver tissue and biomarkers, enzymes and trace elements involved in its metabolism in hatched chicks. Our results showed an inconsistent and dose-dependent toxic effect of ACR on the liver. Despite no changes in most biochemical parameters of the animals' blood, ACR intoxication of chicken embryos, especially at high doses (0.5 mg/egg and 1 mg/egg), leads to negative structural changes in liver tissue among individuals at the time of hatching. Therefore, further studies covering the later postnatal development of animals, other dose ranges or times, as well as the extension of experiments to other research methods are needed. This is very important in order to determine whether the changes induced at the embryonic stage are reversible or whether they will persist throughout the birds' lives. Furthermore, the information obtained on the basis of such extended analyses will constitute valuable research tools contributing not only to the development of toxicology, but also to the protection of poultry health and productivity, as well as to food safety.

## Author Contribution

**Joanna Wojciechowska-Puchałka:** participated in the development of the research concept and methodology, assisted in the process of collecting biological samples and collecting data after hatching; prepared the research material and performed a histological analysis of the liver, conducted a statistical analysis, created figures, and made a formal analysis and interpretation of the results obtained, reviewed the relevant literature, prepared the manuscript, provided the necessary resources and supervised the project's progress, monitored the submission of the manuscript; **Ewa Drąg-Kozak:** performed a trace elements analysis on the liver; reviewed the relevant literature; **Kamil Kustra:** monitored the incubation and injection of eggs, assisted in the process of collecting biological samples and collecting data after hatching; performed a biochemical blood analysis; **Magdalena Trela:** participated in the development of the research concept and methodology; monitored the incubation and injection of eggs, assisted in the process of collecting biological samples and collecting data after hatching, performed a biochemical blood analysis; **Marcin Wojciech Lis:** participated in the development of the research concept and methodology, monitored the incubation and injection of eggs, assisted in the process of collecting biological samples and collecting data after hatching, supervised the realization of the project. All authors have evaluated, read, reviewed, and approved the final version of the manuscript and agree to take personal and public responsibility for all aspects of the work.

## Disclosures

The authors declare no conflict of interest.
